# Prospective European-wide multicentre study on a blood based real-time PCR for the diagnosis of acute schistosomiasis

**DOI:** 10.1186/1471-2334-13-55

**Published:** 2013-01-30

**Authors:** Dominic Wichmann, Sven Poppert, Heidrun Von Thien, Joannes Clerinx, Sebastian Dieckmann, Mogens Jensenius, Philippe Parola, Joachim Richter, Mirjam Schunk, August Stich, Philipp Zanger, Gerd D Burchard, Egbert Tannich

**Affiliations:** 1 Department of Intensive Care Medicine, University Medical Centre Hamburg-Eppendorf, 20246, Hamburg, Germany; 2 Bernhard Nocht Institute for Tropical Medicine, Hamburg, Germany; 3 Department of Clinical Sciences, Institute for Tropical Medicine Antwerp, Antwerp, Belgium; 4 Institute of Tropical Medicine & International Health, Charité Universitätsmedizin Berlin, Berlin, Germany; 5 Department of Infectious Diseases, Oslo University Hospital, Oslo, Norway; 6 Department of Infectious Diseases an Tropical Medicine, Hôpital Nord, Marseille, France; 7 Department of Gastroenterology, Hepatology and Infectious Diseases, Heinrich Heine University Duesseldorf, Duesseldorf, Germany; 8 Department for Infectious Diseases and Tropical Medicine, Ludwig Maximilian University Munich, Munich, Germany; 9 Missionsaerztliche Klinik, Wuerzburg, Germany; 10 Institute of Tropical Medicine, Eberhard Karls University, Tuebingen, Germany; 11 Department of Tropical Medicine, University Medical Centre Hamburg-Eppendorf, Hamburg, Germany

## Abstract

**Background:**

Acute schistosomiasis constitutes a rare but serious condition in individuals experiencing their first prepatent *Schistosoma* infection. To circumvent costly and time-consuming diagnostics, an early and rapid diagnosis is required. So far, classic diagnostic tools such as parasite microscopy or serology lack considerable sensitivity at this early stage of *Schistosoma* infection. To validate the use of a blood based real-time polymerase chain reaction (PCR) test for the detection of *Schistosoma* DNA in patients with acute schistosomiasis who acquired their infection in various endemic regions we conducted a European-wide prospective study in 11 centres specialized in travel medicine and tropical medicine.

**Methods:**

Patients with a history of recent travelling to schistosomiasis endemic regions and freshwater contacts, an episode of fever (body temperature ≥38.5°C) and an absolute or relative eosinophil count of ≥700/μl or 10%, were eligible for participation. PCR testing with DNA extracted from serum was compared with results from serology and microscopy.

**Results:**

Of the 38 patients with acute schistosomiasis included into the study, PCR detected *Schistosoma* DNA in 35 patients at initial presentation (sensitivity 92%). In contrast, sensitivity of serology (enzyme immunoassay and/or immunofluorescence assay) or parasite microscopy was only 70% and 24%, respectively.

**Conclusion:**

For the early diagnosis of acute schistosomiasis, real-time PCR for the detection of schistosoma DNA in serum is more sensitive than classic diagnostic tools such as serology or microscopy, irrespective of the region of infection. Generalization of the results to all Schistosoma species may be difficult as in the study presented here only eggs of S. mansoni were detected by microscopy. A minimum amount of two millilitre of serum is required for sufficient diagnostic accuracy.

## Background

Schistosomiasis is a parasitic infection with trematode flatworms of the genus *Schistosoma*. According to the World Health Organization (WHO) 200 million people in at least 76 countries are affected, most of them located in tropical and subtropical regions [1]. Most infections are attributable to three species: Sc*histosoma mansoni, S. haematobium*, and *S. japonicum*[2]. The courses of human infections can be divided into acute and chronic forms. Acute schistosomiasis, also known as Katayama syndrome [3,4], constitutes a fulminant allergic reaction with fever and eosinophilia and may be accompanied by skin, pulmonary, abdominal or even neurological symptoms. In general, these clinical courses are seen in patients experiencing their first *Schistosoma* infection and reflect an early immunological reaction against developing young worms during prepatency [5]. Accordingly, acute schistosomiasis is usually seen in non-immune travellers returning from *Schistosoma* endemic countries. Considering the broad differential diagnosis of fever and eosinophilia, an early and rapid diagnosis is required to circumvent unnecessary costly and time-consuming diagnostic procedures. However, acute schistosomiasis constitutes a diagnostic challenge as classical tools often fail to detect the parasite at this early stage of infection. Parasite microscopy following Katz-Kato smears or concentration techniques for helminth eggs, lack sensitivity, in particular, in individuals with a low parasite burden and during the prepatent period [6]. In settings with low transmission rates or in patients with low parasite burden immunological tests may be more sensitive than parasite microscopy [7]. Nevertheless, a substantial number of patients remain serologically negative at initial presentation [8,9]. Moreover, the inability of serology to discriminate between active and past disease limits its clinical value for follow up investigations [10]. The detection of *Schistosoma* specific circulating anodic and cathodic antigens (CAA and CCA) in blood or urine, released by viable adult worms, has been tested in schistosomiasis endemic regions and sporadic experiences have been reported for the use in patients with acute schistosomiasis [11-13]. Because the sensitivity of these tests greatly depends on the worm burden, they are probably not suited for the diagnosis of schistosomiasis in travellers with rather low worm burden.

Recently, we reported on the development of a new real-time polymerase chain reaction (PCR) test for the detection of *Schistosoma* specific DNA in blood of infected individuals [14], which revealed excellent performance for the diagnosis of acute schistosomiasis in a small group of travellers returning from Rwanda [15]. Here we report on a European-wide multicenter study to evaluate the use of this PCR for the diagnosis of acute schistosomiasis in larger number of patients returning from various destinations.

## Methods

### Study design

Evaluation of blood based real-time PCR testing in travellers returning from schistosomiasis endemic regions with suspected acute schistosomiasis in 11 specialized European centres in a prospective study from January 2009 to April 2012. In detail the participating countries were Belgium, France, Norway and Germany. The sites are institutions specialised in tropical or travel medicine and are members of the EuroTravNet (http://www.istm.org/eurotravnet/main.html) initiated by The International Society of Travel Medicine and which represents a collaborative network of the European Centre of Disease Control (ECDC). The study was approved by the Hamburg Ethics Committee of the Chamber of Physicians and complied with the Declaration of Helsinki. For the Oslo site data managing files were slightly modified according to local data security requirements, for the other sites no changes were required.

### Inclusion criteria

Patients with a history of a recent travel to a schistosomiasis endemic region and freshwater contacts, an episode of fever (body temperature ≥ 38.5°C) and an absolute or relative eosinophil count of ≥700/μl or ≥10%, were eligible for participation (Group A; n = 38). In addition, 17 patients with high clinical suspect of acute schistosomiasis but without an episode of fever were also tested (Group B). An amount of at least 2 ml of serum was required for DNA extraction and PCR testing as well as for *Schistosoma* serology. Oral informed consent was obtained from every patient or their legal guardians.

### Microscopic parasite detection

Microscopy of faecal or urine samples for the detection of *Schistosoma* eggs were done on individual decision of the attending physician at each centre according to established in-house protocols. In general, stool samples were analysed using the formol-ether concentration technique [16]. For detection of *S. haematobium* eggs, urine collected over a period of 24 hours or at least between 10 am and 2 pm was filtered and processed as described earlier [17].

### Serum and plasma samples

Samples were sent to Hamburg by mail at room temperature and analysed for anti-*Schistosoma* specific antibodies and *Schistosoma* DNA.

### Serology

All patient sera were tested for anti-schistosoma antibodies using an enzyme-immunoassay (EIA) and an immunofluorescence-assay (IFA), respectively [18,19].

### DNA preparation and real time PCR

DNA preparation from 2 ml serum was performed at the Bernhard Nocht Institute for Tropical Medicine, Hamburg, Germany, using the QIAamp Circulating Nucleic Acid Kit according to the manufacturers suggestion (Qiagen, Hilden, Germany). Detection of cell-free *Schistosoma* DNA was performed according to a previously published protocol [14]. The test targets the 121 bp tandem repeat sequence first described by Hamburger et al. [20] using the primer sequences SRA1 (CCACGCTCTCGCAAATAATCT) and SRS2 (CAACCGTTCTATGAAAATCGTTGT) as previously reported [14]. All PCRs were performed in duplicate. A test was considered positive when the threshold was attained within 45 PCR cycles (Ct-value < 45).

### Diagnosis and treatment

A patient was considered positive for acute schistosomiasis when either microscopy, serology or PCR testing revealed a positive result. Treatment with Praziquantel according to local protocols was offered to every patient with a positive test result.

## Results

Thirty-eight patients fulfilled the case definition (Group A). Four of them had a history of possible previous exposure to *Schistosoma*-contaminated water but had not developed symptoms after the first trip. The median time from the day of possible exposure to the onset of clinical symptoms and the median time from exposure to PCR were 35 days (range 12–184 days) and 61 days (range 20–302 days), respectively. Twelve of the patients presented within 6 weeks after the suspected exposure, the time span generally referred to as prepatent stage of *S. mansoni*. The median absolute eosinophil count was 2,290 μl^-1^ (range 870–33,050 μl^-1^). The various members of the study population have been travelled to 17 different African and Arabic countries with endemic schistosomiasis. The median age of the study population was 28 years (range 19 – 66 yr) and 76% were males. Reasons for travelling included: tourism (48%; n = 19), occupational travel (23%; n = 9), volunteer (21%; n = 8) and visiting friends and relatives (8%; n = 3). The patients´ characteristics are summarized in Table 1.

**Table 1:** Characteristics of patients with acute schistosomiasis fulfilling the inclusion criteria

**Patient**	**Region of exposure**	**Reason for travel**	**Incubation time [days]**^**(1)**^	**Time to diagnosis [days]**^**(2)**^	**Eosinophils absolute[per μl]**	**PCR**	**Serology**	**Microscopy**
1	Rwanda	Tourist	57	62	5.400	pos	pos	*S.m.*
2	Rwanda	Tourist	33	54	2.090	pos	(pos)	*S.m.*
3	Mozambique	Occupational	28	42	2.860	pos	pos	Ø
4	Ethiopia	Occupational	27	43	2.650	pos	Ø	Ø
5	Uganda	Occupational	38	56	1.920	pos	pos	Ø
6	Uganda	Occupational	20	32	1.600	pos	pos	n.a.
7	Malawi	Tourist	18	20	1.050	pos	pos	n.a.
8	Mozambique	Tourist	35	56	33.050	pos	Ø	n.a.
9	Yemen	Tourist	40	54	1.540	pos	pos	Ø
10	Malawi	Tourist	27	35	980	pos	Ø	Ø
11^(3)^	Kenya	Tourist	94	96	3.220	Ø	pos	Ø
12	Kenya	Tourist	184	302	1.100	pos	pos	Ø
13	Sudan	Occupational	27	33	3.350	pos	Ø	Ø
14	Tanzania	Volunteer	75	88	5.880	pos	pos	n.a.
15	East Africa	Volunteer	127	131	1.760	pos	pos	Ø
16^(4)^	Kenya	VFR	150	210	n.a.	pos	n.a.	*S.m.*
17	Uganda	Tourist	24	27	n.a.	pos	pos	n.a.
18	Malawi	Tourist	31	73	3.920	pos	Ø	n.a.
19	East Africa	Volunteer	32	104	4.010	pos	pos	Ø
20	Malawi	Tourist	31	34	1.780	Ø	Ø	Ø
21	Mali	Occupational	23	32	1.140	pos	Ø	Ø
22	Tanzania	Volunteer	62	66	2.800	pos	Ø	Ø
23	Madagascar	Tourist	35	38	2.640	pos	pos	n.a.
24	Madagascar	Occupational	92	261	890	pos	pos	n.a.
25	Madagascar	Occupational	122	152	870	pos	pos	Ø
26	East Africa	Tourist	24	36	3.150	pos	pos	Ø
27	Tanzania	Volunteer	47	76	1.700	pos	pos	*S.m.*
28	Tanzania	Volunteer	47	80	9.850	pos	pos	Ø
29	Tanzania	Volunteer	62	94	2.310	pos	pos	Ø
30	Tanzania	Volunteer	97	196	2.290	Ø	pos	Ø
31	Madagsacar	Occupational	n.a.	160	1.100	pos	pos	Ø
32	Madagascar	Tourist	17	43	1.020	pos	pos	*S.m.*
33^(5)^	Ghana	VRF	31	69	1.200	pos	pos	n.a.
34	Madagascar	Tourist	23	34	1.210	pos	pos	n.a.
35	Uganda	Tourist	41	53	2.900	pos	Ø	n.a.
36	Madagascar	Tourist	25	42	23.300	pos	pos	*S.m.*
37	Ghana	Tourist	40	46	1.660	pos	pos	n.a.
38	Tanzania	Tourist	27	119	2.340	pos	pos	n.a.

(1): Time from exposure to beginning of clinical symptoms.

(2): Time from exposure to diagnostics.

(3): Less than 2 ml of Serum was available for DNA extraction.

(4): Patient evaluated for liver transplantation due to known Hepatitis B Virus infection and eosinophilia after visiting her family.

(5): Patient with meningitis and eosinophils in cerebrospinal fluid, Schistosoma DNA detected by PCR in cerebrospinal fluid.

*VFR* Visiting relatives and friends.

*n.a.* Not available.

*pos* Positive.

*Ø* Negative.

*(pos)* Only one of two different serological tests for schistosomiasis shows a positive result.

*S.m.* eggs of *S.mansoni.*

Initial real-time PCR testing revealed positive detection of *Schistosoma* DNA in 35 patients. Of the remaining 3 patients with negative PCR results, the first one was part of a four patients cluster. He had an initial eosinophil count of 2,290 μl^-1^ and a positive serology. The three other patients in this cluster were positive by means of serology and PCR. All received Praziquantel early in the course of the disease and subsequently, laboratory results normalized and clinical symptoms resolved. Accordingly, the negative PCR result in this case was considered as false negative. The second patient with a negative PCR test result was considered true negative, since microscopy and serology remained negative during follow up. The third patient with a negative PCR test result provided only a small amount of serum for PCR testing of less than 2 ml. In favour of a conservative estimation we interpreted this result as false negative because serology was clearly positive and symptoms resolved after Praziquantel treatment. Thus blood based real-time PCR was able to detect acute schistosomiasis in 35 of 37 true positive patients (95%). In contrast, anti-schistosoma antibodies or *Schistosoma* eggs were detected in only 72% and 25%, respectively, of these patients.

For 8 patients follow-up data were available. Four patients presented with negative real-time PCR at their follow up visit. The remaining 4 demonstrated a significant decrease in Ct-values. Median follow-up times of patients with positive PCR test results versus patients with negative PCR test results were 6 and 18 months respectively (data not shown).

In addition, 17 patients (Group B) with high clinical suspect for acute schistosomiasis but without an episode of fever were tested (Table 2). The incubation time and the time from exposure to diagnostic work up was 38 days (range 14–93 days) and 64 days (range 17–96 days), respectively. Sixteen patients were males. The median age was 13 years (range: 6 – 60 yr) and the median eosinophil count was 1,960 μl^-1^ (range 1,040–14,270 μl^-1^). The decision for testing these patients was based on high eosinophil counts, a history of recent travelling to a schistosomiasis endemic region and at least one of the following symptoms: fatigue, gastro-intestinal symptoms, rash or exanthema. Blood based real-time PCR testing was negative in 5 of these patients. In these 5 patients, initial serological testing revealed a positive result in one patient and inconclusive results in two patients. On follow up visits, all of them were serologically negative. Microscopic examination of stool samples was negative in all patients at initial presentation and during follow up. Of the remaining 12 patients, one was returning from Togo with positive serology and microscopy and the other 11 were part of a travel group returning from Rwanda. In this cluster reported earlier [15], PCR was positive in all of them, whereas positive serology or microscopy at initial testing was detected in only 7 of them.

**Table 2:** Characteristics of patients with high suspicion of acute schistosomiasis but without fever

**Patient**	**Region of exposure**	**Reason for travel**	**Incubation time [days]**^**(1)**^	**Time to diagnosis [days]**^**(2)**^	**Eosinophils absolute [per μl]**	**PCR**	**Serology**	**Microscopy**
39	Togo	VFR	51	95	6,000	pos	pos	*S.m.*
40	Brazil	Tourist	39	66	11,640	Ø	Ø	Ø
41	Guinea	Tourist	14	17	1,090	Ø	pos	Ø
42	Uganda	Occupational	38	44	n.a.	Ø	pos	n.a.
43	Egypt	Tourist	26	40	1,100	Ø	Ø	Ø
44	Malawi	Tourist	37	40	1,040	Ø	(pos)	Ø
45	Rwanda	Tourist	47	54	2,640	pos	Ø	*S.m.*
46	Rwanda	Tourist	62	90	14,150	pos	pos	*S.m.*
47	Rwanda	Tourist	57	62	1,150	pos	Ø	Ø
48	Rwanda	Tourist	93	96	2,860	pos	(pos)	*S.m.*
49	Rwanda	Tourist	25	54	14,270	pos	pos	*S.m.*
50	Rwanda	Tourist	n.a.	96	11,120	pos	pos	*S.m.*
51	Rwanda	Tourist	n.a.	96	1,960	pos	pos	Ø
52^(3)^	Rwanda	Tourist	n.a.	62	1,290	pos	Ø	Ø
53^(3)^	Rwanda	Tourist	n.a.	96	1,210	pos	(pos)	*S.m.*
54^(3)^	Rwanda	Tourist	n.a.	96	2,120	pos	(pos)	Ø
55^(3)^	Rwanda	Tourist	n.a.	96	1,700	pos	pos	*S.m.*

(1): Time from exposure to beginning of clinical symptoms.

(2): Time from exposure to diagnostics.

(3): Persons from cluster, exposed 2 years before at the same location as well, and not previously diagnosed.

*VFR* Visiting relatives and friends.

*n.a.* Not available.

*pos* Positive.

*Ø* Negative.

*(pos)* Only one of two different serological tests for schistosomiasis shows a positive result.

*S.m.* eggs of *S.mansoni.*

## Discussion

To our knowledge this is the first multicenter study on acute schistosomiasis in travellers residing in non-endemic countries. Moreover, it constitutes the largest prospective study on a blood based real-time PCR for the diagnosis of the disease. The high rate of positive PCR results within the study population may be in part attributed to the fact that the participating centres are well familiar with this otherwise rare disease of acute schistosomiasis as all centres are specialized in travel and tropical medicine.

The results presented here support previous findings that PCR for the detection of *Schistosoma* DNA in serum outperforms other diagnostic test such as serology or microscopy in the early phase of the infection, particular in the prepatent stage of human schistosomiasis [14], as demonstrated by the twelve patients presenting within 42 days after the suspected exposure. By PCR, a correct diagnosis was made in 95% of patients, despite the heterogeneous characteristics of the study population (17 endemic countries - cluster and single patients), which underlines the robustness of blood base real-time PCR testing for the diagnosis of acute schistosomiasis. In addition, PCR was able to exclude active *Schistosoma* infections in six patients, of whom 50% were suspected due to positive serology (1 positive, 2 inconclusive). Thus, the results support previous observations that the PCR test system used in this study is highly sensitive and specific [14]. Accordingly, this test system has substantial added value for an early and rapid decision making to circumvent cost-intensive and time-consuming diagnostic procedures and to prevent unnecessary therapies. Cost for one PCR test (materials and reagents) are roughly 2 US$, thus the test is a useful addition in the differential diagnosis in patients with fever and eosinophilia after travelling to schistosomiasis endemic countries but is not generally recommended as a screening assay for returning travelers.

A considerable disadvantage of classical *Schistosoma* diagnostics is the persistence of serologic markers and egg shedding after successful treatment. Previous studies in experimentally infected mice [14] suggested that blood based real-time PCR testing might be suitable not only for initial diagnosis but also for follow up monitoring. This is further supported by our findings in 8 patients, for whom follow up data were available. These results suggest a significant decline in Ct-values in response to treatment. Clearly negative PCRs are expected after a longer period of time due to the high sensitivity of PCR and the carry over of DNA from slowly degenerating eggs in the tissue. However, for a definite conclusion on the usefulness of PCR for monitoring of schistosomiasis treatment the follow-up of a larger number of patients is required.

The highly repetitive 121 bp DNA fragment used as target sequence in the real-time PCR assay constitutes about 12% of the *S. mansoni* genome [20]. Although this sequence is detected in *S. haematobium* and *S. japonicum* as well [20,21] sensitivity of PCR for the latter two species might be reduced. As far as species could be determined by microscopic egg morphology, the vast majority of cases in this study were due to infections by *S. mansoni.* Thus, our results may not allow generalization to infections with *Schistosoma* species other than *S. mansoni.* Evaluation of blood based PCR for the diagnosis of acute schistosomiasis due to other *Schistosoma* species such as *S. haematobium* or *S. japonicum* requires further studies. Previous results with the assay used in this study indicated that the performance of the PCR using the 121 bp tandem target sequence on 2 ml serum samples works very well in *S. mansoni* infection, but not in *S. haematobium* and in *S. mekongi* infection (Jan Clerinx, personal communication). Likewise, additional studies are required to evaluate the usefulness of blood-based PCR for the diagnosis of chronic schistosomiasis, in particular, in suspected cases from endemic countries, with a positive serology but negative parasite microscopy. Moreover, recent studies on, PCR performed with urine or fecal samples to detect *S. haematobium* or *S. mansoni* infection have shown rather promising results [22-25]. However, further studies are required to determine, whether urine and/or feces PCR is sensitive to detect early schistosoma infection as it is the case in patients with acute schistosomiasis.

## Conclusion

For the early diagnosis of acute schistosomiasis, real-time PCR for the detection of *Schistosoma* DNA in serum is more sensitive than classic diagnostic tools such as serology or microscopy, irrespective of the region of infection. Generalization of the results to all *Schistosoma* species may be difficult as in the study presented here only eggs of *S. mansoni* were detected by microscopy. A minimum amount of two millilitre of serum is required for sufficient diagnostic accuracy.

## Competing interests

All authors declare to have no competing interests.

## Authors’ contribution

GDB, ET and DW designed the study protocol. JC, SD, MJ, PP, JR, MS, AS, PS, GDB and DW coordinated the study at their local institution, including collection of patients data and clinical specimens. SV, JC, SD, MJ, PP, JR, MS, AS, PZ and ET were responsible for performing serological tests, analysis and interpretation of the data. SV, HvT and ET were responsible for performing PCR tests, analysis and interpretation of the data. SV, JC, ET and DW performed the statistical analysis. SV, ET and DW drafted the manuscript. JC and PP account for the critical revision of the manuscript. All authors have given approval to the final version of the manuscript.

## Pre-publication history

The pre-publication history for this paper can be accessed here:

http://www.biomedcentral.com/1471-2334/13/55/prepub
